# Low Oral Bioavailability and Partial Gut Microbiotic and Phase II Metabolism of Brussels/Witloof Chicory Sesquiterpene Lactones in Healthy Humans

**DOI:** 10.3390/nu12123675

**Published:** 2020-11-28

**Authors:** Hui Weng, Luanying He, Jiakun Zheng, Qing Li, Xiuping Liu, Dongliang Wang

**Affiliations:** 1 Department of Nutrition, School of Public Health, Sun Yat-sen University (Northern Campus), Guangzhou 510080, China; wengh3@mail2.sysu.edu.cn (H.W.); hely29@mail2.sysu.edu.cn (L.H.); zhengjk3@mail2.sysu.edu.cn (J.Z.); liqing85@mail2.sysu.edu.cn (Q.L.); liuxp26@mail2.sysu.edu.cn (X.L.); 2 Guangdong Provincial Key Laboratory of Food, Nutrition and Health, Guangzhou 510080, China; 3 Guangdong Engineering Technology Research Center of Nutrition Translation, Guangzhou 510080, China

**Keywords:** bioavailability, Brussels/witloof chicory, gut microbiotic metabolism, phase II metabolism, pharmacokinetics, sesquiterpene lactones

## Abstract

Free and glycosylated sesquiterpene lactones (SLs), which are abundant in leafy vegetables including Brussels/witloof chicory, possess health-promoting effects in vivo. However, the pharmacokinetics of dietary source of SLs remain largely unknown. In this open-label and single-dose trial, sixteen healthy volunteers consumed 150 g of Brussels/witloof chicory juice containing 48.77 μmol SLs in 5 min. Blood, urine, and fecal samples were collected before and after chicory consumption in 24 h. No SLs were detected in the serum, urine, and fecal samples before chicory consumption in all of the participants. Chicory consumption increased lactucin, 11β,13-dihydrolactucin, and their glucuronide/sulfate conjugates, rather than lactucopicrin and 11β,13-dihydrolactucopicrin, as well as glycosylated SLs in biological samples. The peak concentration of total SLs in serum reached 284.46 nmol/L at 1 h, while, in urine, this peak was 220.3 nmol between 2 and 6 h. The recovery of total SLs in blood, urine, and feces was 7.03%, 1.13%, and 43.76% of the ingested dose, respectively. Human fecal suspensions with intestinal microbiota degraded glycosylated SLs in chicory, and converted lactucopicrin and 11β,13-dihydrolactucopicrin to lactucin and 11β,13-dihydrolactucin, respectively. Collectively, Brussels/witloof chicory SLs are poorly bioavailable and they undergo partial gut microbial and phase II metabolism in humans.

## 1. Introduction

Sesquiterpene lactones (SLs) are a colorless, bitter, and stable subfamily of terpenes [1]. Over 5000 structures of SLs are widely distributed within the plant kingdom, most of which are derived from the family Asteraceae [2]. Lettuce and chicory (*Lactuca sativa* and *Chicorium intybus* L.) that belong to the family Asteraceae are the leading sources of SLs in the human diet [3]. The principal constitutive SLs in lettuce and chicory are lactucin, 11β,13-dihydrolactucin, lactucopicrin (esterified lactucin), and 11β,13-dihydrolactucopicrin (esterified 11β,13-dihydrolactucin), in either free or glycosylated form [4,5].

Lactucin, lactucopicrin, and 11β,13-dihydrolactucin have been reported to possess considerable analgesic and sedative activities in mice [6]. These capacities were comparable to ibuprofen, which is a widely used nonsteroidal anti-inflammatory drug [6]. We recently found that SLs-rich Brussels/witloof chicory (*Chicorium intybus* L. *var*. *foliosum*), one variety of chicory, is able to slow down the progression of established atherosclerosis in mice [7,8]. More importantly, SLs-rich chicory and lettuce have been used in traditional medicines globally for centuries [9,10]. Last but not least, several in vitro cell culture studies have shown that lactucin or lactucopicrin possesses appreciable biological activities, such as anti-malarial [11], anti-cancer [12,13], and neuroprotection [14,15]. Overall, SLs-rich foods might be considered to be part of a healthy, balanced diet.

Illustration of the bioavailability and metabolism of any exogenous compounds is fundamental in understanding their impacts on health states in vivo. Very recently, García et al. found, for the first time, that lactucin and lactucopicrin from curly escarole, one leafy vegetable from the family Asteraceae, are orally bioavailable and undergo gut microbiotic metabolism (dehydroxylation and reduction) and phase II metabolism (glucuronidation and sulfation) in healthy humans [16], as evidenced by a qualitative analysis of SLs and these metabolites in urine. However, the significance of this pharmacokinetic study might be limited by a lack of quantification for lactucin and lactucopicrin in curly escarole, a few participants (three males and two females), absence of quantification for lactucin and lactucopicrin, and these metabolites in the urine, blood, and feces, as well as no dietary restrictions in order to ensure that lactucin and lactucopicrin during the intervention were derived from curly escarole alone. Moreover, the fate of glycosylated SLs in curly escarole were not traced after these oral consumption.

In the current study, we used both qualitative and quantitative approaches to evaluate the absorption, metabolism, and excretion of SLs in sixteen healthy humans after oral intake of Brussels/witloof chicory. Meanwhile, the metabolism of SLs from Brussels/witloof chicory by human fecal suspensions in the presence or absence of intestinal microbiota was investigated.

## 2. Materials and Methods

### 2.1. Materials

Brussels/witloof chicory juice was handmade by the homogenization of 2700 g of fresh Brussels/witloof chicory (Hebei Vilof Agritech Co., Ltd., Beijing, China) in 1800 mL of water while using a juice making machine (HAIPAN, Model BL-6A, Zhongshan, China). Lactucin, 11β,13-dihydrolactucin, lactucopicrin, and 11β,13-dihydrolactucopicrin were purchased from Extrasynthese (Lyon-Nord, France). Methanol was used to prepare stock solutions of standard SLs. Working standard solutions were prepared daily by dilution in methanol. Sulfatase (type H-1 from *Helix* pomatia, containing β-glucuronidase), β-glucuronidase (EC 3.2.1.31, type IX A from E. *coli*), d-saccharic acid 1,4-lactone (a glucuronidase inhibitor), santonin, and brain heart infusion broth were from Sigma–Aldrich (St. Louis, MO, USA). The cellulase enzyme (from *Aspergillus niger*) was from Solarbio (Beijing, China).

### 2.2. Clinical Pharmacokinetic Study

A detailed description of the clinical pharmacokinetic study in eight male and eight female healthy participants (body mass index, 19–24.7 kg·m^−2^; aged 27–51 years) was recently published [17]. Briefly, each participant after an overnight fast (>12 h) drank up a single dose of Brussels/witloof chicory juice containing 150 g of Brussels/witloof chicory in 5 min. All of the participants provided blood (baseline, 0.5, 1, 2, 4, 6, 12, 24 h), urine (baseline, individual voids between 0 and 2 h, 2 and 6 h, 6 and 12 h, and 12–24 h), and fecal samples (baseline, all voids between 0 and 24 h). The volume of urine and weight of feces excreted was measured, and aliquots were stored at −80 °C before analysis by using high-performance liquid chromatography (HPLC). Of note, all of the participants followed a diet free of vegetables, fruits, and plant-based beverages for the day before the intervention and the intervention day, which were to limit any residual dietary SL compounds from the body and also ensure that SL compounds that were present in biological samples collected during the intervention came from Brussels/witloof chicory juice alone. The study was registered at http://www.chictr.org.cn/showproj.aspx?proj=24307 as ChiCTR1800014393.

### 2.3. Sample Preparation

Free and glycosylated SLs in Brussels/witloof chicory were isolated and analyzed, as previously described [18]. Briefly, 0.5 g of freeze-dried Brussels/witloof juice powder were mixed with the internal standard (0.41 μmol of santonin). The sample was than extracted twice by 2% (*v*/*v*) formic acid in methanol/water 4/1 (*v*/*v*). All of the supernatants were dried by nitrogen flow, recovered with methanol, and then divided into two parts. One part was used to quantify free SLs, and another underwent an enzymatic hydrolysis with cellulase enzyme (activity: 0.8 units/mg) to convert glycosylated SLs into their free forms (to catalyze the hydrolysis of glycosidic bonds in order to remove sugars from glycosylated SLs) [4]. Both free and glycosylated SL-containing fractions were purified from phenols and interfering compounds by solid phase extraction, employing Silica cartridges (Bond Elut SI cartridge, 500 mg, 3 mL) from Agilent Technologies (Santa Clara, CA, USA). Briefly, after conditioning and equilibrating the solid phase extraction cartridges, the samples were loaded and eluted with dichloromethane/ethyl acetate 3/2 (*v*/*v*). The loading and elution fractions were collected, evaporated under vacuum at 35 °C, and then recovered with methanol/water 1/1 (*v*/*v*) before high-performance liquid chromatography (HPLC) analysis.

The serum, urine, and fecal samples from each participant were prepared, as previously described [19]. In brief, aliquots of homogeneous serum (600 μL), urine (600 μL) or fecal (0.5 g) samples were spiked with the internal standard (0.24 nmol of santonin) and treated according to one of the four following procedures: no treatment (to detect free SLs); cellulase (to detect glycosylated SLs); β-glucuronidase (to detect glucuronidated and sulfated SLs); or, sulfatase in the presence of d-saccharic acid 1,4-lactone (to detect sulfated SLs) [18,19,20]. The levels of glycosylated and sulfated SLs were indirectly calculated by subtracting free form of SLs in the raw materials (Brussels/witloof chicory, serum, urine, or feces) from those in the cellulase- and sulfatase-treated materials, respectively. The levels of glucuronidated SLs were indirectly calculated by subtracting SLs in the sulfatase-treated materials (serum, urine, or feces) from those in the β-glucuronidase. Thereafter, 2400 μL of methanol was added to 600 μL serum or urine sample in a sealed glass vial. 3 mL of normal saline and methanol (1:3, *v*/*v*) was then added to 0.5 g fecal sample. After vortexing, the sample vials were centrifuged at 14,000 rpm for 10 min. The serum, urine, or fecal supernatant was collected and purified by solid phase extraction before HPLC analysis. Of note, the use of santonin was required for accurate quantification, because it permitted taking the losses due to SLs extraction and further analytical treatments into account [18].

### 2.4. HPLC Method

SLs in Brussels/witloof chicory and biological samples were measured by a HPLC assay, as previously described with minor modifications [18]. HPLC analyses were carried out on a Waters 2695 Alliance HPLC (Waters Corp., Milford, MA, USA) with a photodiode array detector (mod. 2998). SL elution was carried out in gradient mode while employing the following solvent system: mobile phase A: methanol/water 14/86 (*v*/*v*); mobile phase B: methanol. The gradient program was as follows: from 0 to 20 min, 100–58% A; from 20 to 30 min, 58–42% A; from 30 to 45 min, 42–10% A; from 45 to 46 min, 0–100% A; and, from 46 to 55 min, 100% A as post-run. The flow rate was 0.5 mL/min, and the injection volume was 20 μL. The data were processed by the software Empower (ver. 3.0) from Waters. Each chromatogram was recorded at 260 nm, whereas the absorption spectra were recorded between 200 and 400 nm. The column was a Zorbax SB-C18 (250 mm × 4.6 mm, 5.0 μm) column from Agilent Technologies. The analyses were performed at 25 °C. For each analytical run, a standard curve was prepared in the appropriate matrix (methanol, blank serum, urine, or feces) and used to quantify the content of SL in chicory or biological samples. Standard curves were constructed from the peak area ratios of the analyte to internal standard (santonin) versus analyte concentrations while using a 1/x^2^ weighted linear least-squares regression model. The detection limit for lactucin, 11β,13-dihydrolactucin, lactucopicrin, and 11β,13-dihydrolactucopicrin (10-fold baseline noise) under the conditions used in this study was between 9.06 and 12.59 nmol/L in methanol, 9.06 and 14.39 nmol/L in serum, 9.06 and 12.59 nmol/L in urine, and 10.87 and 12.59 nmol/L in feces, and values that were below this concentration were reported as zero. Of note, recoveries of SL were higher than 82.4% in methanol, serum, urine, and feces. The intra- and inter-day variations were below 9.52% in all sample types. Santonin was selected as the internal standard.

### 2.5. Pharmacokinetic Analysis

The pharmacokinetic parameters of SLs in the free, glucuronide, and sulfate conjugates were calculated while using DAS 2.1 (BioGuider Co., Shanghai, China) with a noncompartmental model: maximum serum concentrations (C_max_); time to achieve maximum serum concentrations (T_max_); the area under the concentration-time curve to 24 h (AUC_0–24_); and, terminal elimination half-life (T_1/2z_). The recovery of total SLs in the blood was calculated while using AUC_0-24_ divided by their ingested dose (all SLs in Brussels/witloof chicory). The recovery of total SLs in urine and feces was calculated using the content of urinary and fecal SLs divided by their ingested dose (all SLs in Brussels/witloof chicory).

### 2.6. Catabolism of SLs by Human Fecal Suspensions

The in vitro fermentation of lactucopicrin, 11β,13-dihydrolactucopicrin, or Brussels/witloof chicory SLs by human fecal suspensions with or without intestinal microbiota was carried out as we have previously described for the fermentation of Brussels/witloof chicory phenolic acids [17]. In brief, fresh fecal samples (2 g) that were collected from a healthy female volunteer aged 19 who had not consumed fruits, vegetables and plant-based beverages/wine for 48 h were mixed with 10 mL of sterile brain heart infusion broth to prepare human fecal suspensions. The human fecal suspensions (100 μL) with or without heat treatment were then incubated with 1 μmol of lactucopicrin, 11β,13-dihydrolactucopicrin, or freeze-dried Brussels/witloof chicory powder (0.14 g) at 37 °C under anaerobic conditions. The samples were collected after 0, 0.5, 1, 2, and 24 h of fermentation, followed by an acidification with 30 μL of 6 M HCl to inactivate the microbiota and enzymes before being stored at −80 °C. Concordantly, the human fecal suspensions without any incubation with SLs or Brussels/witloof chicory were considered to be a negative control.

### 2.7. Statistical Analysis

The data are presented as the means ± SEM. The significance of differences between the baseline (0 h) and the indicated time points was assessed by ANOVA for repeated measures and the Dunnett’s 2-tailed *t* test, while assuming the baseline values as the reference category. *p* < 0.05 was considered to be statistically significant.

## 3. Results

### 3.1. SLs in Brussels/Witloof Chicory

**Figure 1:**
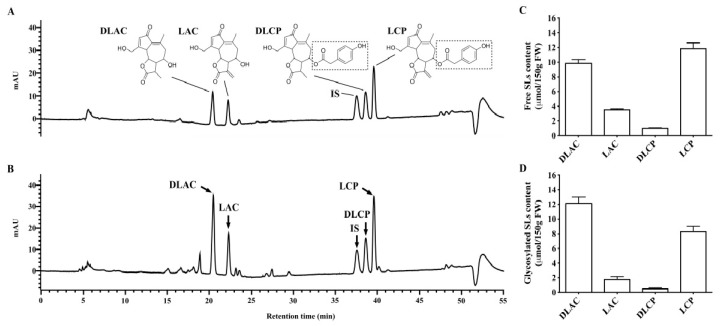
Characterization and quantification of sesquiterpene lactones in Brussels/witloof chicory. (**A**,**B**) Representative chromatographic profiles of sesquiterpene lactones in Brussels/witloof chicory treated without (**A**) or with cellulase (**B**). Santonin was served as an internal standard. (**C**,**D**) The content of free (**C**) or glycosylated (**D**) sesquiterpene lactones from 150 g of Brussels/witloof chicory. Values are the means ± SEM, *n* = 6. SLs, sesquiterpene lactones; LAC, lactucin; DLAC, 11β,13-dihydrolactucin; LCP, lactucopicrin; DLCP, 11β,13-dihydrolactucopicrin; IS, internal standard; FW, fresh weight. Dotted box, ester of 4-hydroxyphenylacetic acid.

Representative HPLC chromatograms of free or glycosylated SL extracts from Brussels/witloof chicory are shown in Figure 1A,B, respectively. Figure 1C,D—present the content of free and glycosylated SLs (lactucin, 11β,13-dihydrolactucin, lactucopicrin, and 11β,13-dihydrolactucopicrin) in the Brussels/witloof chicory juice portion (150 g of Brussels/witloof chicory) offered to each participant. 11β,13-Dihydrolactucin represented most of the SLs (45.04%), and lactucin contributed 10.8%, 11β,13-dihydrolactucopicrin 2.99%, and lactucopicrin 41.2%. The total amount of SLs in the 150 g of Brussels/witloof chicory was 48.8 μmol. Moreover, the content of SLs in the free and glycosylated forms were comparable in Brussels/witloof chicory (Figure 1C,D).

### 3.2. SLs and Metabolites in Serum, Urine and Feces

At baseline, lactucin, 11β,13-dihydrolactucin, lactucopicrin, and 11β,13-dihydrolactucopicrin in the free, glycosylated, and their corresponding glucuronide/sulfate conjugated forms in serum, urine, and feces were not detectable in all participants. These data suggest that all of the participants had not consumed foods containing SLs before Brussels/witloof chicory consumption.

**Figure 2:**
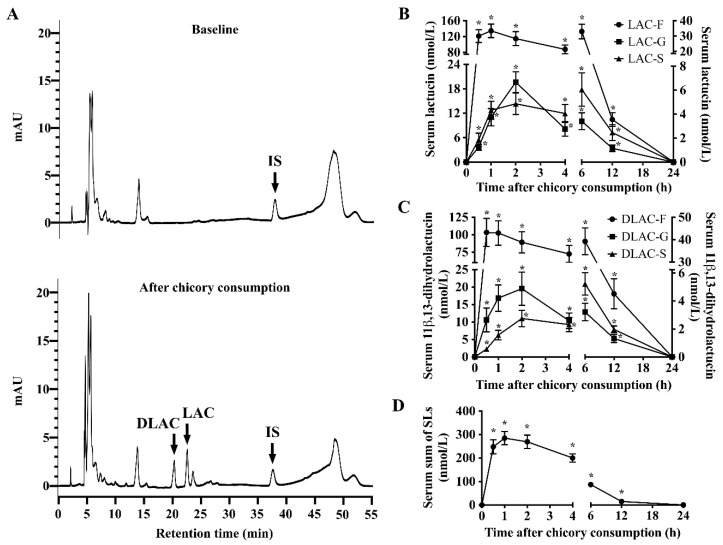
Characterization and quantification of serum concentrations of sesquiterpene lactones and metabolites over 24 h in 16 healthy adults after Brussels/witloof chicory consumption. (**A**) Representative chromatographic profiles of free sesquiterpene lactones in human serum sample collected at baseline (**A**, top) or at 1 h after the consumption of Brussels/witloof chicory (**A**, bottom). (**B**–**D**) Serum concentration-time curves (0–24 h) of free and glucuronide/sulfate conjugated LAC (**B**), DLAC (**C**), and the sum total of sesquiterpene lactones (**D**). Values are the means ± SEM, *n* = 16. * Different from baseline, *p* < 0.05. SLs, sesquiterpene lactones; LAC, lactucin; DLAC, 11β,13-dihydrolactucin; IS, internal standard; F, free; G, glucuronide; S, sulfate.

Strikingly, Brussels/witloof chicory consumption increased the levels of free lactucin, 11β,13-dihydrolactucin, and their corresponding glucuronide/sulfate conjugated forms in the serum of all participants (Figure 2A and Supplementary Materials Figure S1). Moreover, lactucopicrin and 11β,13-dihydrolactucopicrin and their glucuronide/sulfate conjugates, as well as the glycosylated forms of all SLs, were not detectable (Figure 2A and Supplementary Materials Figure S1). The serum concentration-time curve of free lactucin and 11β,13-dihydrolactucin showed for their compounds a C_max_ of 143 ± 17.9 and 116 ± 19.5 nmol/L at 0.88 and 0.78 h post consumption, respectively (Figure 2B,C; Table 1). The peak concentration of total SLs in serum reached 284.46 nmol/L at 1 h (Figure 2D). For serum lactucin in its glucuronided and sulfated form, T_max_ was 2.06 h and 2.81 h, respectively. For 11β,13-dihydrolactucin in its glucuronided and sulfated form, T_max_ was 2.69 h and 2.38 h, respectively (Table 1). For serum free, glucuronide, and sulfate conjugated forms of lactucin, T_1/2z_ was 2.13 h, 2.80 h, and 4.14 h, respectively (Table 1). For serum free, glucuronide and sulfate conjugated forms of 11β,13-dihydrolactucin, T_1/2z_ was 2.53 h, 3.17 h, and 5.01 h, respectively (Table 1).

**Figure 3:**
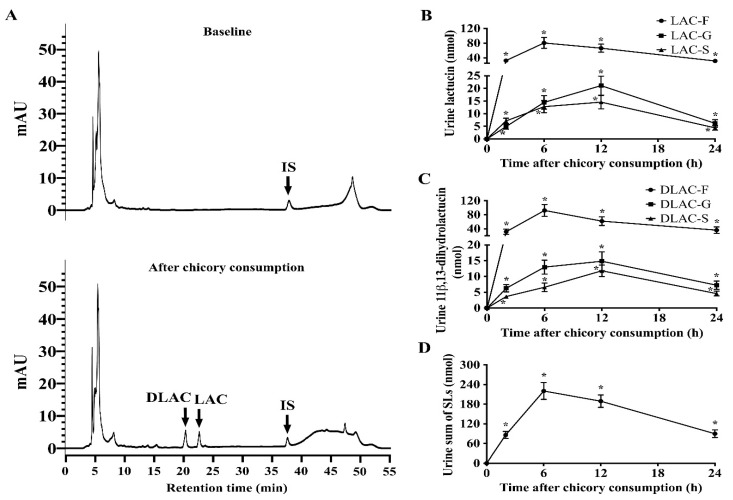
Urine contents of sesquiterpene lactones and metabolites over 24 h in 16 healthy adults after Brussels/witloof chicory consumption. (**A**) Representative chromatographic profiles of free sesquiterpene lactones in human urine sample collected at baseline (**A**, top) or at 2–6 h after the consumption of Brussels/witloof chicory (**A**, bottom). (**B**–**D**) Urine excretion of free and glucuronide/sulfate conjugated LAC (**B**), DLAC (**C**), and the sum total of sesquiterpene lactones (**D**). Values are the means ± SEM, *n* = 16. * Different from baseline, *p* < 0.05. SLs, sesquiterpene lactones; LAC, lactucin; DLAC, 11β,13-dihydrolactucin; IS, internal standard; F, free; G, glucuronide; S, sulfate.

Regarding the urine, Brussels/witloof chicory consumption increased free lactucin, 11β,13-dihydrolactucin, and their corresponding glucuronide and sulfate conjugates (Figure 3A–C and Supplementary Materials Figure S2). Moreover, free and glucuronide/sulfate conjugated lactucopicrin and 11β,13-dihydrolactucopicrin and the glycosylated forms of all SLs were not detectable in urine (Supplementary Materials Figure S2). The excretion of total SLs (lactucin and 11β,13-dihydrolactucin) reached a plateau from 2–6 h after Brussels/witloof chicory consumption (Figure 3D).

**Figure 4:**
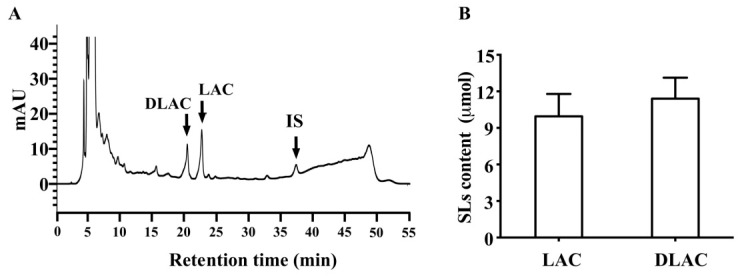
Characterization of sesquiterpene lactones in feces after Brussels/witloof chicory consumption. (**A**) Representative chromatographic profiles of free sesquiterpene lactones in human fecal sample collected in 24 h after the consumption of Brussels/witloof chicory. (**B**) Content of total sesquiterpene lactones in human fecal sample collected in 24 h after the consumption of Brussels/witloof chicory. LAC, lactucin; DLAC, 11β,13-dihydrolactucin; IS, internal standard.

Brussels/witloof chicory consumption increased lactucin and 11β,13-dihydrolactucin in the free form in feces, as shown in Figure 4A. Moreover, glucuronide/sulfate conjugated lactucin and 11β,13-dihydrolactucin, lactucopicrin, and 11β,13-dihydrolactucopicrin, as well as the glycosylated forms of all SLs, were not detectable in all participants. The mean contents of free lactucin and 11β,13-dihydrolactucin were 9.95 ± 1.84 μmol and 11.4 ± 1.72 μmol (Figure 4B), respectively.

### 3.3. Recovery of SLs and Metabolites in Serum, Urine and Feces

The recovery of total SLs from the blood, urine, and feces was approximately 7.03%, 1.13%, and 43.8% of the consumption dose (Table 2), respectively. Moreover, the glucuronide and sulfate conjugates accounted for about 10.6% and 11.0% of total SLs in the blood, and 14.9% and 11.2% of total SLs in urine (Table 2), respectively.

### 3.4. Catabolism of SLs by Human Fecal Suspensions

Lactucopicrin and 11β,13-dihydrolactucopicrin were incubated with fecal suspensions with or without heat treatment. While fecal suspension incubation resulted in the complete disappearance of lactucopicrin and 11β,13-dihydrolactucopicrin in 2 h, lactucin and 11β,13-dihydrolactucin were generated (Table 3). However, heat-treated fecal suspensions did not affect the stability of lactucopicrin and 11β,13-dihydrolactucopicrin withinin 24 h, and lactucin and 11β,13-dihydrolactucin were not generated (Table 3). In addition, fecal suspensions degraded lactucopicrin and 11β,13-dihydrolactucopicrin, as well as the glycosylated forms of all SLs from Brussels/witloof chicory to undetectable levels within 2 h (Table 4), while this was not the case for heat-treated fecal suspensions. These data implied that human fecal suspensions could degrade esterified and glycosylated SLs from Brussels/witloof chicory into their corresponding free aglycones.

## 4. Discussion

Understanding the pharmacokinetics of SLs from the human diet is a key step in dissecting their potential health-promoting effects in vivo. García et al. recently showed that lactucin and lactucopicrin from curly escarole are orally bioavailable and they undergo gut microbiotic metabolism (dehydroxylation and reduction) and phase II metabolism (glucuronidation and sulfation) in healthy humans [16]. However, this study was based on a qualitative analysis of SLs and these metabolites in urine in five healthy adults. Herein, using both qualitative and quantitative approaches we evaluated the pharmacokinetics of SLs in sixteen healthy adults over a 24-h period after a single oral consumption of 150 g of Brussels/witloof chicory. We obtained three major findings: (1) lactucin, 11β,13-dihydrolactucin, and their glucuronide/sulfate conjugates, rather than lactucopicrin and 11β,13-dihydrolactucopicrin, as well as all glycosylated SLs, were detectable in serum, urine, and fecal samples; (2) the peak concentration of total SLs in serum reached 284 nmol/L at 1 h, while, in urine, this peak was 220 nmol between 2 and 6 h; and, (3) the recovery of total SLs that mainly exist as the free form in the systemic circulation, urine and feces was 7.03%, 1.13% and 43.8% of the consumption dose, respectively. In addition, our in vitro fermentation assays showed that human fecal suspensions with intestinal microbiota degraded all glycosylated SLs in Brussels/witloof chicory and converted lactucopicrin and 11β,13-dihydrolactucopicrin to lactucin and 11β,13-dihydrolactucin, respectively. Collectively, together with the qualitative findings from the pharmacokinetics of curly escarole SLs [16], our findings allow us for to propose that Brussels/witloof chicory SLs are likely to be poorly absorbed and they undergo partial gut microbial and phase II metabolism in humans. Whether the poorly bioavailable SLs from Brussels/witloof chicory elicit pharmacological/biological activities in humans are worth further investigation.

The absence of lactucopicrin (esterified lactucin) and 11β,13-dihydrolactucopicrin (esterified 11β,13-dihydrolactucin), as well as all glycosylated SLs in serum, urine, and feces in humans after Brussels/witloof chicory consumption, prompted us to dissect the potential mechanisms. Esterases and glycosidases that are secreted by intestinal microbiota and/or intestinal mucosa are known to efficiently hydrolyze the ester or glycoside bond of plant secondary metabolites to their corresponding free aglycones, respectively [21,22]. Indeed, esterases that are secreted by intestinal microbiota and/or intestinal mucosa have been reported to release the 4-hydroxylated phenyl-acetaldehyde in lactucopicrin from curly escarole [16]. Therefore, we conducted in vitro fermentation assays in which SLs were incubated with fresh human fecal suspensions with or without heat treatment. Consistent with the in vivo findings, lactucopicrin was undetectable in fresh human fecal suspensions without heat treatment, whereas lactucin was generated, which accounted for 45.2% of its parent lactucopicrin. In contrast, lactucopicrin was stable in fresh human fecal suspensions with heat treatment, where lactucin was undetectable. Similar to the conversion from lactucopicrin to lactucin, fresh human fecal suspensions also converted 11β,13-dihydrolactucopicrin to 11β,13-dihydrolactucin. We incubated glycosylated SLs from Brussels/witloof chicory with fresh human fecal suspensions in order to test whether glycosidases secreted by intestinal microbiota and/or intestinal mucosa can degrade glycosylated SLs in Brussels/witloof chicory. Strikingly, fresh human fecal suspensions degraded all of the glycosylated SLs from Brussels/witloof chicory in 2 h to undetectable levels. This degradation was not due to their spontaneous degradation in human fecal suspensions, because all glycosylated SLs were stable in heated-treated fresh human fecal suspensions. Moreover, the amount of lactucin and 11β,13-dihydrolactucin was far higher than those naturally occurring in Brussels/witloof chicory, supporting the notion that glycosidases that are secreted by intestinal microbiota and/or intestinal mucosa degrade glycosylated SLs in Brussels/witloof chicory. Together, these in vitro findings suggested that esterases and glycosidases that are secreted by intestinal microbiota/intestinal mucosa are likely responsible for the absence of lactucopicrin and 11β,13-dihydrolactucopicrin, as well as all glycosylated SLs in humans after consumption of Brussels/witloof chicory.

Plant secondary metabolites with one or more hydroxyl groups typically undergo an extensive phase-II metabolism in animals and humans. Consistently, we recently found that, after a single oral intake of Brussels/witloof chicory, the recovery of protocatechuic acid (one specific phenolic acid) in its glucuronidated and sulfated forms in the blood circulation, urine. and feces was 34.8%, 60.2%, and 72.7%, respectively [17]. Unlike protocatechuic acid, the recovery of Brussels/witloof chicory SLs in their glucuronidated and sulfated forms in the blood circulation, urine, and feces were all below 26.1%. The mechanism underlying this obvious difference in the phase II metabolism between protocatechuic acid and SLs remains unknown. One possible explanation might be a competitive pathway for protocatechuic acid and SLs to undergo glucuronidation and sulfation in humans, as the amount of protocatechuic acid in Brussels/witloof chicory is over four-fold higher than that of SLs. Other phenolic acids with hydroxyl groups (e.g., gallic, caffeic, 5-caffeoylquinic, caftaric, and chicoric acid) are also constitutes of Brussels/witloof chicory [19], which might further compete with SLs. Moreover, lactucin and lactucopicrin from curly escarole could be dehydroxylated by human gut microbiota [16]. Thus, it is logical to hypothesize that dehydroxylation of SLs from Brussels/witloof chicory is another explanation for the less phase II metabolism of SLs than that of protocatechuic acid.

Although 52.0% of SLs from Brussels/witloof chicory have been recovered in the systemic circulation, urine, and feces, the other was undetected in humans. We favored the following four potential explanations. First, because SLs from curly escarole undergo gut microbiota metabolism (dehydroxylation and reduction) and isomerization in humans [16], the undetected 48.0% of SLs from Brussels/witloof chicory may be partially or fully due to those dehydroxylated, reduced, and isomerized metabolites that are not measured in this study. Second, because goat gut microbiota is able to extensively degrade SLs [23,24], it is also possible that human gut microbiota degraded SLs from Brussels/witloof chicory into these metabolites that are not monitored in the current studies. Indeed, our in vitro fermentation assays showed that only 60.7% of SLs were recovered in fresh human fecal suspensions, whereas 89.3% of SLs were recovered in heat-treated human fecal suspensions. Third, the pharmacokinetic studies of other SLs, such as isoalantolactone and alantolactone, showed that these compounds are distributed extensively in the liver of rats [19]. Thus, it is possible that the undetected 48.0% of SLs from Brussels/witloof chicory results from their distribution in the liver of humans. Fourth, the higher recovery of total SLs from Brussels/witloof chicory in blood (7.03% of the ingested dose) than that in urine (1.13% of the ingested dose) suggest that the absorbed compounds are extensively distributed into organs/tissues in general, and adipose tissues in particular, as SLs and these gut microbiota metabolites are nonpolar or weakly polar compounds.

We and others have consistently shown that human gut microbiota could convert SLs into these metabolites [16]. For example, human gut microbiota could release the 4-hydroxylated phenyl-acetaldehyde in both lactucopicrin and 11β,13-dihydrolactucopicrin. However, there was only one peak of SLs in the systemic circulation that occurred at 1 h after Brussels/witloof chicory consumption, which suggests that most, if not all, SLs or these gut microbiota metabolites are likely to be poorly absorbed or even unabosrbed in the lower part of intestinal tract.

## 5. Conclusions

We provided evidence that SLs from Brussels/witloof chicory are likely to be poorly absorbed and undergo partial gut microbial and phase II metabolism in humans. Because SLs possess appreciable pharmacological effects in preclinical models, it is of interest to investigate whether Brussels/witloof chicory and other SLs-rich foods (e.g, curly escarole, lettuce, and radicchio) recapitulate their effects in humans.

## Figures and Tables

**Table 1:** Pharmacokinetic characteristics of sesquiterpene lactones and metabolites in serum over 24 h in 16 healthy adults after Brussels/witloof chicory consumption ^a^.

Compound	C_max_ (nmol·L^−1^)	T_max_ (h)	T_1/2z_ (h)	AUC_0–24_ (nmol·h·L^−1^)
LAC-F	142 ± 17.9	0.88 ± 0.12	2.13 ± 0.22	649 ± 84.3
LAC-G	19.8 ± 2.54	2.06 ± 0.14	2.80 ± 0.23	71.6 ± 10.8
LAC-S	16.3 ± 2.65	2.81 ± 0.31	4.14 ± 0.38	87.1 ± 14.5
DLAC-F	116 ± 19.5	0.78 ± 0.10	2.53 ± 0.22	563 ± 96.3
DLAC-G	22.1 ± 4.44	2.69 ± 0.30	3.17 ± 0.37	83.3 ± 15.2
DLAC-S	13.2 ± 2.17	2.38 ± 0.29	5.01 ± 0.55	67.1 ± 8.87

^a^ Values are the means ± SEM, *n* = 16; LAC, lactucin; DLAC, 11β,13-dihydrolactucin; F, free; G, glucuronide; S, sulfate; C_max_, maximum serum concentrations; T_max_, time to achieve maximum serum concentrations; T_1/2z_, terminal elimination half-life; AUC_0–24_, the area under the concentration-time curve to 24 h.

**Table 2:** Recovery of total sesquiterpene lactones in blood, urine, and feces over 24 h in 16 healthy adults after Brussels/witloof chicory consumption ^a^.

Total Sesquiterpene Lactones	Recovery (of % Oral Consumption)		
	Serum	Urine	Feces
Free	5.51 ± 0.13	0.83 ± 0.03	43.75 ± 4.34
Glucuronide	0.75 ± 0.07	0.17 ± 0.02	ND
Sulfate	0.78 ± 0.11	0.13 ± 0.02	ND

^a^ Values are the means ± SEM, *n* = 16; Total sesquiterpene lactones were defined by the quantification of lactucin and 11β,13-dihydrolactucin in either free, glucuronidated, or sulfated form; ND, not detected.

**Table 3:** Mean content of sesquiterpene lactones detected in active or heat-inactivated fecal suspensions incubated with lactucopicrin or 11β,13-dihydrolactucopicrin ^a^.

Compound (μmol)	Incubation Time (h) with Active Fecal Suspensions					Incubation Time (h) with Heat-Inactivated Fecal Suspensions				
	0	0.5	1	2	24	0	0.5	1	2	24
LCP	0.84 ± 0.04	0.28 ± 0.07 *	0.21 ± 0.10 *	ND	ND	0.86 ± 0.03	0.91 ± 0.07	0.88 ± 0.08	0.86 ± 0.06	0.81 ± 0.12
*LAC* ^b^	ND	0.43 ± 0.16 *	0.48 ± 0.13 *	0.59 ± 0.17 *	0.38 ± 0.14 *	ND	ND	ND	ND	ND
DLCP	0.89 ± 0.05	0.34 ± 0.08 *	0.16 ± 0.11 *	ND	ND	0.91 ± 0.06	0.90 ± 0.05	0.84 ± 0.15	0.85 ± 0.11	0.83 ± 0.09
*DLAC* ^b^	ND	0.39 ± 0.15 *	0.53 ± 0.12 *	0.62 ± 0.14 *	0.44 ± 0.16 *	ND	ND	ND	ND	ND

^a^ Values are the means ± SEM, *n* = 5; LAC, lactucin; DLAC, 11β,13-dihydrolactucin; LCP, lactucopicrin; DLCP, 11β,13-dihydrolactucopicrin; ND, not detected. ^b^ Metabolite generated from lactucopicrin or 11β,13-dihydrolactucopicrin by human fecal suspensions. * *p* < 0.05 compared with the *T*_0_ groups.

**Table 4:** Mean content of sesquiterpene lactones detected in active or heat-inactivated fecal suspensions incubated with Brussels/witloof chicory ^a^.

Compound (nmol)	Incubation Time (h) with Active Fecal Suspensions					Incubation Time (h) with Heat-Inactivated Fecal Suspensions				
	0	0.5	1	2	24	0	0.5	1	2	24
LAC	50.4 ± 2.84	89.0 ± 14.6 *	147 ± 32.7 *	211 ± 64.1 *	159 ± 43.0 *	48.3 ± 2.07	42.0 ± 7.49	46.3 ± 6.82	42.2 ± 6.07	45.0 ± 10.3
DLAC	158 ± 7.43	274 ± 46.1 *	435 ± 111 *	514 ± 132 *	384 ± 91.1 *	154 ± 5.52	143 ± 24.4	157 ± 41.61	141 ± 13.6	135.47 ± 19.3
LCP	211 ± 13.3	183 ± 28.9	127 ± 20.6 *	ND	ND	200 ± 10.2	209 ± 17.7	184 ± 29.4	199 ± 31.6	204 ± 27.8
DLCP	17.2 ± 1.14	9.76 ± 1.67 *	4.37 ± 1.04 *	ND	ND	18.1 ± 0.98	16.5 ±1.53	16.3 ± 1.28	16.6 ± 4.12	17.3 ± 3.61
LAC-Gly	46.1 ± 3.77	37.8 ± 4.57	13.2 ± 5.93 *	ND	ND	48.2 ± 2.9	44.9 ± 9.47	42.6 ± 9.78	39.7 ± 8.58	42.0 ± 5.68
DLAC-Gly	249 ± 18.0	184 ± 26.0 *	57.2 ± 17.8 *	ND	ND	238 ± 15.78	226 ± 29.2	219 ± 25.5	209 ± 46	205 ± 43.2
LCP-Gly	153 ± 9.13	55.1 ± 14.1 *	9.91 ± 2.1*	ND	ND	149 ± 12.0	134 ± 25.6	124 ± 20.0	128 ± 15.3	127 ± 26.9
DLCP-Gly	9.04 ± 1.07	5.67 ± 1.19 *	1.47 ± 0.75 *	ND	ND	9.2 ± 1.13	8.46 ± 1.35	8.04 ± 1.21	8.19 ± 1.14	7.84 ± 1.36

^a^ Values are the means ± SEM, *n* = 5; LAC, lactucin; DLAC, 11β,13-dihydrolactucin; LCP, lactucopicrin; DLCP, 11β,13-dihydrolactucopicrin; Gly, glycosylated form; ND, not detected. * *p* < 0.05 compared with the *T*_0_ groups.

## Supplementary Materials

The following are available online at https://www.mdpi.com/2072-6643/12/12/3675/s1, Figure S1: Characterization of sesquiterpene lactones in serum after Brussels/witloof chicory consumption, representative chromatographic profiles of (**A**) glucuronidated, (**B**) sulfated or (**C**) glycosylated sesquiterpene lactones in human serum sample collected at 1 h after the consumption of Brussels/witloof chicory. LAC, lactucin; DLAC, 11β,13-dihydrolactucin; IS, internal standard, Figure S2: Characterization of sesquiterpene lactones in urine after Brussels/witloof chicory consumption, representative chromatographic profiles of (**A**) glucuronidated, (**B**) sulfated or (**C**) glycosylated sesquiterpene lactones in human urine sample collected at 2–6 h after the consumption of Brussels/witloof chicory. LAC, lactucin; DLAC, 11β,13-dihydrolactucin; IS, internal standard.

## References

[1] Ghantous A., Gali-Muhtasib H., Vuorela H., Saliba N.A., Darwiche N. (2010). What made sesquiterpene lactones reach cancer clinical trials?. Drug Discov. Today.

[2] Amorim M.H., Gil D.C.R., Lopes C., Bastos M.M. (2013). Sesquiterpene lactones: Adverse health effects and toxicity mechanisms. Crit. Rev. Toxicol..

[3] Chadwick M., Trewin H., Gawthrop F., Wagstaff C. (2013). Sesquiterpenoids lactones: Benefits to plants and people. Int. J. Mol. Sci..

[4] Price K.R., Dupont M.S., Shepherd R., Chan H.W.S., Fenwick G.R. (1990). Relationship between the Chemical and Sensory Properties of Exotic Salad Crops -Coloured Lettuce (Lactuca sativa) and Chicory (Cichorium intybus. J. Sci. Food Agric..

[5] Wulfkuehler S., Gras C., Carle R. (2014). Influence of light exposure during storage on the content of sesquiterpene lactones and photosynthetic pigments in witloof chicory (*Cichorium intybus* L. var. foliosum Hegi). LWT Food Sci. Technol..

[6] Wesolowska A., Nikiforuk A., Michalska K., Kisiel W., Chojnacka-Wojcik E. (2006). Analgesic and sedative activities of lactucin and some lactucin-like guaianolides in mice. J. Ethnopharmacol..

[7] Lin W., Liu C., Yang H., Wang W., Ling W., Wang D. (2015). Chicory, a typical vegetable in Mediterranean diet, exerts a therapeutic role in established atherosclerosis in apolipoprotein E-deficient mice. Mol. Nutr. Food Res..

[8] Liu C., Wang W., Lin W., Ling W., Wang D. (2016). Established atherosclerosis might be a prerequisite for chicory and its constituent protocatechuic acid to promote endothelium-dependent vasodilation in mice. Mol. Nutr. Food Res..

[9] Street R.A., Sidana J., Prinsloo G. (2013). Cichorium intybus: Traditional Uses, Phytochemistry, Pharmacology, and Toxicology. Evid. Based Complement. Altern..

[10] Nwafor I.C., Shale K., Achilonu M.C. (2017). Chemical Composition and Nutritive Benefits of Chicory (Cichorium intybus) as an Ideal Complementary and/or Alternative Livestock Feed Supplement. Sci. World J..

[11] Bischoff T.A., Kelley C.J., Karchesy Y., Laurantos M., Nguyen-Dinh P., Arefi A.G. (2004). Antimalarial activity of lactucin and lactucopicrin: Sesquiterpene lactones isolated from *Cichorium intybus* L.. J. Ethnopharmacol..

[12] Ren Y.L., Zhou Y.W., Chen X.Z., Ye Y.H. (2005). Discovery, structural determination and anticancer activities of lactucin-like guaianolides. Lett. Drug Des. Discov..

[13] Zhang F.H., Yan Y.L., Wang Y., Liu Z. (2016). Lactucin induces potent anti-cancer effects in HL-60 human leukemia cancer cells by inducing apoptosis and sub-G1 cell cycle arrest. Bangl. J. Pharmacol..

[14] Venkatesan R., Subedi L., Yeo E.J., Kim S.Y. (2016). Lactucopicrin ameliorates oxidative stress mediated by scopolamine-induced neurotoxicity through activation of the NRF2 pathway. Neurochem. Int..

[15] Venkatesan R., Shim W.S., Yeo E.J., Kim S.Y. (2017). Lactucopicrin potentiates neuritogenesis and neurotrophic effects by regulating Ca(2+)/CaMKII/ATF1 signaling pathway. J. Ethnopharmacol..

[16] Garcia C.J., Beltran D., Tomas-Barberan F.A. (2020). Human Gut Microbiota Metabolism of Dietary Sesquiterpene Lactones: Untargeted Metabolomics Study of Lactucopicrin and Lactucin Conversion In Vitro and In Vivo. Mol. Nutr. Food Res..

[17] Zheng J., Xiong H., Li Q., He L., Weng H., Ling W., Wang D. (2019). Protocatechuic acid from chicory is bioavailable and undergoes partial glucuronidation and sulfation in healthy humans. Food Sci. Nutr..

[18] Ferioli F., D’Antuono L.F. (2012). An update procedure for an effective and simultaneous extraction of sesquiterpene lactones and phenolics from chicory. Food Chem..

[19] Xu R., Zhou G., Peng Y., Wang M., Li X. (2015). Pharmacokinetics, tissue distribution and excretion of isoalantolactone and alantolactone in rats after oral administration of Radix Inulae extract. Molecules.

[20] Shelnutt S.R., Cimino C.O., Wiggins P.A., Ronis M.J., Badger T.M. (2002). Pharmacokinetics of the glucuronide and sulfate conjugates of genistein and daidzein in men and women after consumption of a soy beverage. Am. J. Clin. Nutr..

[21] Andreasen M.F., Kroon P.A., Williamson G., Garcia-Conesa M.T. (2001). Esterase activity able to hydrolyze dietary antioxidant hydroxycinnamates is distributed along the intestine of mammals. J. Agric. Food Chem..

[22] Gonthier M.P., Remesy C., Scalbert A., Cheynier V., Souquet J.M., Poutanen K., Aura A.M. (2006). Microbial metabolism of caffeic acid and its esters chlorogenic and caftaric acids by human faecal microbiota in vitro. Biomed. Pharmacother..

[23] Broudiscou L., Cornu A., Rouzeau A. (2007). In vitro degradation of 10 mono- and sesquiterpenes of plant origin by caprine rumen micro-organisms. J. Sci. Food Agric..

[24] Malecky M., Albarello H., Broudiscou L.P. (2012). Degradation of terpenes and terpenoids from Mediterranean rangelands by mixed rumen bacteria in vitro. Animal.

