# Supplementary material for: Low Oral Bioavailability and Partial Gut Microbiotic and Phase II Metabolism of Brussels/Witloof Chicory Sesquiterpene Lactones in Healthy Humans
Source: Nutrients. 2020 Nov 28;12(12):3675. doi: 10.3390/nu12123675 (PMC7760865; doi:10.3390/nu12123675)
Supplement: Supplementary file 1 [file nutrients-12-03675-s001.pdf]

Supplemental materials

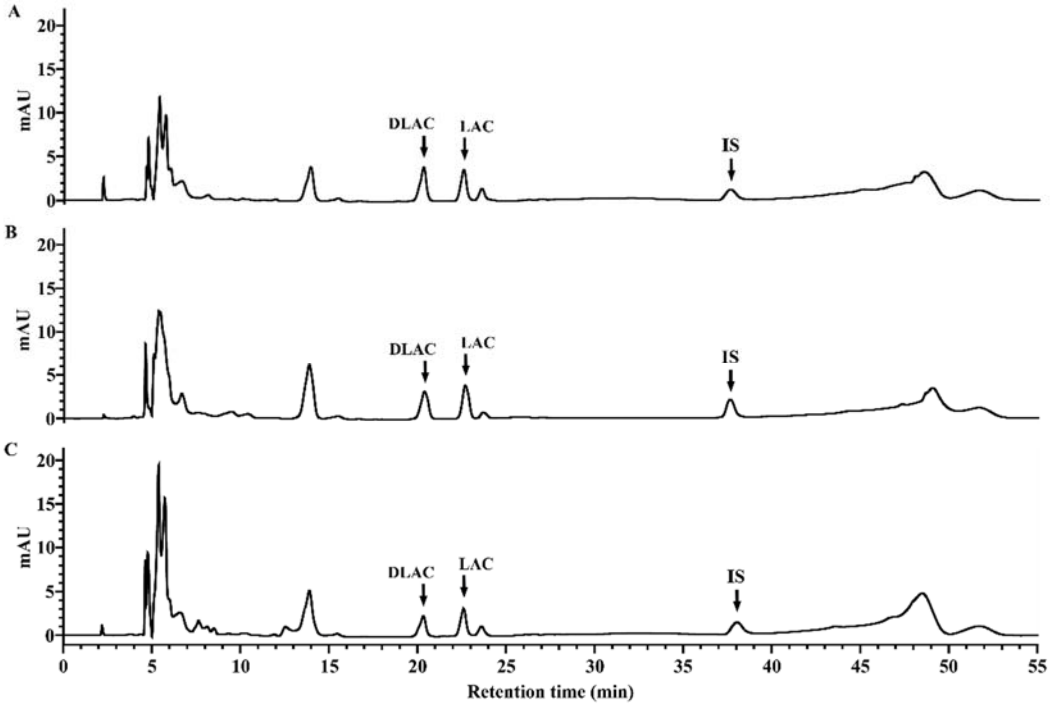

Figure S1. Characterization of sesquiterpene lactones in serum after Brussels/witloof chicory consumption. Representative chromatographic profiles of (**A**) glucuronidated, (**B**) sulfated or (**C**) glycosylated sesquiterpene lactones in human serum sample collected at 1 h after the consumption of Brussels/witloof chicory. LAC, lactucin; DLAC, 11β,13-dihydrolactucin; IS, internal standard.

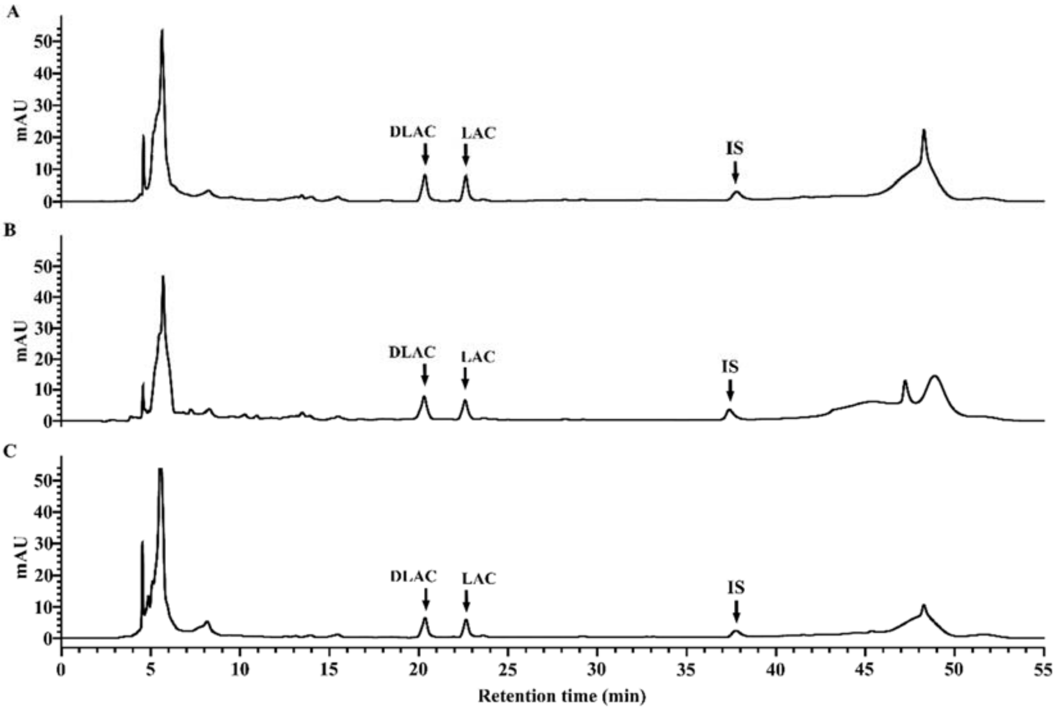

Figure S2. Characterization of sesquiterpene lactones in urine after Brussels/witloof chicory consumption. Representative chromatographic profiles of (**A**) glucuronidated, (**B**) sulfated or (**C**) glycosylated sesquiterpene lactones in human urine sample collected at 2-6 h after the consumption of Brussels/witloof chicory. LAC, lactucin; DLAC, 11β,13-dihydrolactucin; IS, internal standard.
